# Second-Line Treatment Options for Small-Cell Lung Cancer: A Light at the End of the Tunnel

**DOI:** 10.3390/cancers16020255

**Published:** 2024-01-05

**Authors:** Fausto Meriggi

**Affiliations:** Oncology Department, Istituto Ospedaliero Fondazione Poliambulanza, Via Leonida Bissolati 57, 25124 Brescia, Italy; famerigg@libero.it or fausto.meriggi@poliambulanza.it

**Keywords:** extensive-stage small-cell lung cancer, second-line treatments, biology and targeted therapies, new agents

## Abstract

**Simple Summary:**

Small-cell lung cancer accounts for approximately 15% of all lung cancers and for about 30 years it has relied on the same chemo- and radiotherapeutic treatment strategies. Recently, there have been new promising and effective options introduced in the first-line treatment of the extensive stage of the disease, such as immunotherapy with atezolizumab or durvalumab added to standard chemotherapy, and moreover in heavily pretreated patients with new agents such as tarlatamab. This review aims to provide an overview of the current therapeutic strategies in the second-line treatment of small-cell lung cancer with the hope that we will finally begin to see a light at the end of the tunnel.

**Abstract:**

Small-cell lung cancer (SCLC) is a subtype of lung tumor characterized by rapid growth and early metastatic dissemination. It represents approximately 15% of all diagnosed lung cancers, with an annual incidence of over 200,000 cases worldwide. At the time of initial diagnosis, approximately 75–80% of patients already have extrathoracic spread. Almost all patients with SCLC also relapse after achieving a complete response with first-line treatment. Outcomes achievable in second-line treatment are related to the length of time between completion of first-line therapy and disease progression. While first-line chemo-immunotherapy remains the standard of care for initial management, the role of second-line treatment strategies in SCLC has been a topic of significant research and discussion. Second-line treatment options are limited and the results are still disappointing. Several molecules are currently being studied in lines following the first, using immunological targets and cell cycle checkpoints. Among these, particular interest has been placed on anti-PD-1 (programmed cell death-1 protein) and anti-PD-L1 (programmed cell death-ligand 1) monoclonal antibodies, and DLL3 (Delta-like ligand 3), which are being evaluated alone or in combination. Tarlatamab is a novel promising therapeutic antibody currently under investigation for its potential use in previously treated SCLC patients. This mini-review will explore the current state of second-line treatment options for SCLC, their clinical efficacy, and future directions.

## 1. Introduction

SCLC is a highly aggressive form of lung cancer, accounting for approximately 15% of all lung cancer cases with an annual incidence of over 200,000 cases worldwide [1]. In particular, more than 90% of patients affected by SCLC are men, of advanced age (70 and above), current or former heavy smokers, and with cardiopulmonary or metabolic comorbidities. SCLC originates from neuroendocrine cells present in the bronchi and usually develops in the central thoracic position [2]. It is characterized by rapid growth and early metastasis, making it one of the most lethal tumors. The majority of patients with SCLC are diagnosed at an extensive stage of the disease (ED), where treatment options become limited. At the time of initial diagnosis, approximately 75–80% of patients already have extrathoracic spread (to other organs), preferentially to the brain, liver, adrenal glands, bones, or bone marrow. Although first-line chemo-immunotherapy remains the standard of care for initial management [3,4], almost all patients with SCLC also relapse after achieving remission with first-line treatment. Therefore, the role of second-line treatment strategies in SCLC has been the subject of significant research and discussion.

## 2. First-Line Treatments

The first-line treatment in ED SCLC has been platinum-containing combination chemotherapy for decades. A randomised phase III study that included patients with limited-stage disease (LD), the combination of cisplatin and etoposide was shown to be superior to the popular three-drug combination containing anthracycline (cyclophosphamide + epirubicin + vincristine) [5]. The results of the study favored the combination of cisplatin and etoposide, with a significant prolongation of median survival. Additionally, the percentage of patients alive at 2 and 5 years was significantly better in the cisplatin- and etoposide-treated arm in the overall population. However, in a subgroup analysis based on disease stage, the combination of cisplatin and etoposide was found to be superior in the subgroup of patients with LD, while no significant differences were found in patients with ED.

Afterwards, several randomized studies compared the combination of cisplatin and etoposide with another combination in which cisplatin was accompanied by a different drug than etoposide.

In 2002, the results of a randomized phase III study conducted in Japan [6], comparing the combination of cisplatin and irinotecan with the standard combination of cisplatin and etoposide, generated significant interest. The experimental treatment was found to be superior to the cisplatin + etoposide combination in terms of overall survival (OS), and the difference (12.8 versus 9.4 months) was statistically significant. Unfortunately, as also occurred in other studies conducted on other diseases, the results obtained in the Asian population in favor of the irinotecan combination were not confirmed in two similar studies conducted in the United States [7,8]. Moreover, a meta-analysis of 12 randomized studies did not show differences in efficacy between the two treatment regimens [9].

Several randomized studies have compared cisplatin-containing regimens with carboplatin-containing regimens in the treatment of SCLC without reporting any substantial advantage for a specific regimen used [10,11]. A meta-analysis conducted with individual patient data, totaling 663 patients, did not show differences in terms of OS (HR 1.08, 95% CI 0.92–1.27; *p* = 0.37) and progression-free survival (PFS) (HR 1.10, 95% CI 0.94–1.29; *p* = 0.25) between cisplatin-based and carboplatin-based regimens, respectively. The difference was reported in terms of toxicity, as carboplatin-containing regimens were found to be more myelotoxic, while cisplatin-containing regimens were burdened by greater non-hematological toxicity [12].

Recently, immunotherapy (IO) has been carefully studied in ED SCLC [13]. In the first-line therapy of ED, the addition of atezolizumab (anti-PD-L1) to standard chemotherapy with platinum salts and etoposide in combination for four cycles, followed by maintenance with atezolizumab, has shown an increase in OS of 2 months and PFS of 0.9 months in the phase III IMpower133 study, regardless of the PD-L1 value [3]. A similar result was obtained in the first-line therapy with the addition of durvalumab in combination with standard chemotherapy, followed by maintenance with durvalumab, achieving an increase in OS of 2.7 months in the phase III CASPIAN study [4]. The results of both studies are statistically significant and, despite the magnitude of the benefit in OS being modest, they represent the first positive result with an increase in survival for SCLC after over 30 years. Conversely, the KEYNOTE 604 study, with pembrolizumab in combination with platinum and etoposide, failed to demonstrate a survival benefit compared to chemotherapy alone. However, the signal in favor of the pembrolizumab combination was strong [14], and also in a clinical scenario that frequently requires the concomitant administration of high doses of steroids.

Patients who maintain a good PS without having a bulky disease usually receive radiation therapy (RT) that is integrated with chemotherapy early. However, RT is frequently postponed after the third cycle of chemotherapy in cases of patients with poor PS and/or with a bulky disease. This approach has the advantage of reducing myelosuppression and loco-regional toxicities.

## 3. Second-Line Treatments

**Figure 1:**
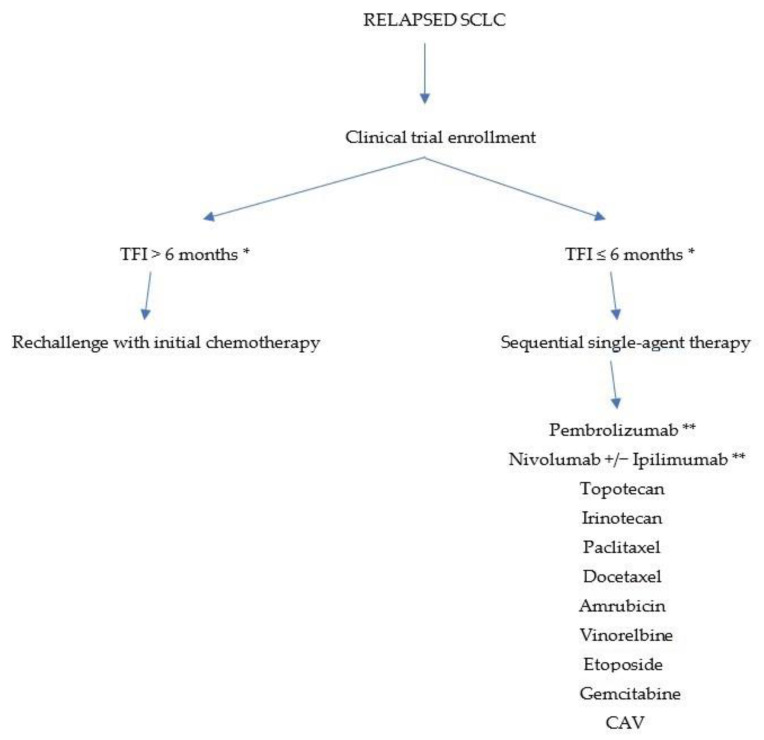
Treatment algorithm for relapsed SCLC. TFI—treatment-free interval after initial therapy; CAV—Cyclophopsphamide, Doxorubicin, Vincristine; * if clinical trial enrollment is not possible; ** strategies including PD-1 agents with or without anti-CTLA-4 are no longer of real interest since the introduction of PD-L1 agents in first-line chemo-immunotherapy.

Almost all patients with SCLC experience a relapse after achieving a complete response to first-line treatment. The outcomes achievable in the second line are related to the time period between the completion of first-line therapy and disease progression. Based on this parameter, patients with relapsed SCLC can be divided into “resistant”, with a progression-free interval of less than 3 months from the end of therapy until progression; “sensitive” when they have a long duration of response to previous therapy; and “refractory” when disease progression occurs during first-line treatment. For “resistant” patients, the chances of response to treatment are very low; for “sensitive” patients, especially if the interval since the completion of first-line treatment exceeds 6 months, the use of the same first-line chemotherapy (i.e., a “rechallenge”) may be considered, but it should be emphasized that this possibility is not unequivocally supported by solid evidence; for “refractory” patients, the benefit of salvage therapy remains very uncertain (Figure 1). Overall, relapsed patients have a poor prognosis, with a median survival of 2–3 months without further treatment and about 6 months in case of response to second-line therapy [15].

The first randomized phase III trial conducted on 211 patients with sensitive SCLC after an initial response to first-line therapy compared intravenous (IV) topotecan with a three-drug combination regimen (cyclophosphamide, doxorubicin, and vincristine: CAV). The study included the enrollment of patients who experienced progression at least 60 days after previous treatment. Topotecan, administered at a dose of 1.5 mg/m^2^ per day for 5 days every 3 weeks, resulted in an objective response rate in 24.3% of cases compared to 18.3% reported with polychemotherapy (*p* = 0.285). However, this difference was not statistically significant as the results were also similar in terms of median time to progression (TTP) (13.3 versus 12.3 weeks, respectively) and OS (25 vs. 24.7 weeks, respectively). Regarding toxicity, grade 4 neutropenia was statistically more frequent with polychemotherapy, while grade 3–4 anemia and thrombocytopenia were statistically more frequent with topotecan. The study also evaluated the impact of therapy on at least eight symptoms related to lung disease, and topotecan was statistically significantly superior to polychemotherapy in four symptoms, including dyspnea, anorexia, asthenia, and dysphonia [16].

Another randomized phase III trial compared oral topotecan (at a dose of 2.3 mg/m^2^ per day for 5 days every 3 weeks) with supportive care alone in 141 patients who experienced progression after first-line treatment and were deemed unsuitable for IV treatment. Oral topotecan resulted in a significant increase in OS (25.9 vs. 13.9 weeks, *p* = 0.0104), a slower deterioration of quality of life (QoL), and better symptom control compared to supportive care alone, at the cost of predictable hematological toxicity. It should be noted that a significant advantage in favor of topotecan was also observed in the subgroup of patients with a worse prognosis, namely those with a short interval since the end of first-line chemotherapy [17]. Subsequently, in a phase III study, the two formulations of topotecan, IV and oral at standard doses, were compared in the treatment of 309 patients with relapsed SCLC and a progression-free interval of at least 90 days. OS was 32 weeks with oral topotecan and 35 weeks with the IV formulation. The toxicity profile showed a higher incidence of thrombocytopenia and diarrhea with the oral formulation and a higher incidence of anemia with IV topotecan [18]. Based on the aforementioned results, topotecan, both in the IV and oral formulations, is the only drug that has been registered for the treatment of patients with SCLC progressing after first-line treatment (Table 1).

Noteworthy is a recent phase III study which demonstrates comparable results in platinum-sensitive patients treated with topotecan or rechallenge with carboplatin and etoposide [19].

In second-line therapy for SCLC, Amrubicin (A), a new synthetic anthracycline, has also been evaluated. Several phase II studies, including randomized ones, have shown that A is active and relatively well-tolerated in this group of patients [20,21]. However, most of this study was conducted on Asian patients. A phase III randomized trial comparing A with topotecan in 673 Western patients with sensitive or refractory SCLC to first-line treatment did not show any differences in OS for A compared to topotecan, but only a better response rate (RR) (31% vs. 17%). A survival advantage of A was reported in the 295 refractory SCLC patients (6.2 versus 5.7 months; *p* = 0.047), corresponding to an absolute advantage of only 15 days [22,23].

Recently, the activity of the alkylating drug Lurbinectedin (L) was evaluated in a phase II study that included 105 patients with ED SCLC in second-line treatment, with a median PFS of 3.9 months. In the subgroup of patients with a chemotherapy-free interval of more than 90 days (defined as “sensitive”), the median PFS was 4.6 months, with a 6-month PFS rate of 44.6% [24], and it received approval from both the FDA and EMA for this indication. The combination of L and doxorubicin was evaluated in the phase III ATLANTIS trial, which failed to demonstrate an OS advantage compared to the CAV regimen [25].

Other molecules are currently being studied in lines following the first, using immunological targets and cell cycle checkpoints. Among these, particular interest has been placed on anti-PD-1 and anti-PD-L1 antibodies that work by blocking the proteins that inhibit the body’s immune system from attacking cancer cells [26,27], and on anti-DLL3, which are being evaluated alone or in combination. DLL3 is a protein that is specifically overexpressed in SCLC cells and which promotes cell proliferation and survival. Targeting DLL3 with an antibody can potentially disrupt this pathway and inhibit the growth of cancer cells [28].

In lines following the first, two anti-PD-1 antibodies, nivolumab (alone or plus ipilimumab) and pembrolizumab, have so far shown encouraging results in phase I and II studies, with RR of up to 19% and a good tolerability profile [26,27] (Table 1). However, despite initial FDA approval, the respective companies have recently withdrawn the indication for both immunotherapeutic agents as monotherapies in second-line treatment for ED SCLC. It is important to note that while ICIs have shown promise, they are not effective for all SCLC patients. As seen in several trials of immunotherapy, the benefit of OS is not really represented by a major increase in median OS, but in the tails of the curves. In the SCLC space, there are approximately 10% more patients in the immunotherapy arms that benefit in the long-term scale (3 years and more). RR is generally higher in patients with high PD-L1 expression, and further research is needed to identify predictive biomarkers and improve patient selection [3].

**Table 1:** Main clinical trials of 2nd-line chemotherapy and immunotherapy in relapsed SCLC.

Design	Agents	RR (%)	mPFS (Months)	mOS (Months)	Author (Ref.)
Phase III 2nd-line (positive)	Topotecan IV vs. CAV	24.3 vs. 18.3	13.3 vs. 12.3 wks	25 vs. 24.7 wks	Von Pawel [16]
Phase III 2nd-line (positive)	Topotecan PO vs. BSC	7 vs. NR	16.3 wks vs. NR	25.9 vs. 13.9 wks	O’Brien [17]
Phase III 2nd-line (positive)	PO vs. IV Topotecan	18.3 vs. 21.9	11.9 vs. 14.6 wks	33.0 vs. 35.0 wks	Eckardt [18]
Phase III 2nd-line (negative; positive in refractory SCLC patients)	Amrubicin vs. Topotecan IV	31.1 vs. 16.9	4.1 vs. 3.5	7.5 vs. 7.8 Refractory: 6.2 vs. 5.7 (*p* 0.047)	Von Pawel [23]
Phase I/II ≥1 prior line of therapy (positive)	Nivolumab 3 mg/kg vs. Nivolumab 1 mg/kg plus Ipilimumab 3 mg/kg vs. Nivolumab 3 mg/kg plus Ipilimumab 1 mg/kg	10.0 vs. 23.0 vs. 19.0	1.4 (95% Cl, 1.4 to 1.9) vs. 2.6 (95% CI, 1.4 to 4.1) vs. 1.4 (95% Cl, 1.3 to 2.2	4.7 (95% Cl, 3.0 to 9.3) vs. 7.7 (95% Cl, 3.6 to NR) vs. 7.2195% Cl, 3.7 to NR)	Antonia [26]
Phase Ib ≥2 prior line of therapy (positive)	Pembrolizumab 10 mg/kg every 2 wks or 200 mg every 3 wks	19.3	NR (61% of responders 18+)	NE	Chung [27]
Phase II 2nd-line (positive)	Lurbinectedin 3.2 mg/mq every 3 wks	35.2	NE	NE	Trigo [24]
Phase II ≥2 prior line of therapy (positive)	Tarlatamab 10 mg or 100 mg IV every 2 wks	40 vs. 32	4.9 vs. 3.9	Estimated OS at 9 months: 68% vs. 66%	Ahn [28]

IV—intravenous; CAV—Cyclophopsphamide, Doxorubicin, Vincristine; PO—per os; BSC—best supportive care; CI—confidence interval; NR—not reached; NE—not evaluable; wks—weeks.

Tarlatamab (T) is a novel therapeutic bi-specific antibody engaging immune response currently under investigation for its potential use in the treatment of SCLC. T acts as an antibody by targeting and binding to DLL3, a protein that is specifically expressed in SCLC cells. By binding to DLL3, T aims to inhibit the growth and survival of SCLC cells while sparing healthy cells with low or no expression of DLL3. Early preclinical studies have shown promising results, suggesting that T can effectively kill SCLC cells both in vitro (in laboratory cultures) and in vivo (in animal models). These findings have sparked further investigation into its potential as a therapeutic option for SCLC.

Recently, in a phase II study consisting of 220 patients with previously treated SCLC (patients had received a median of two lines of treatment), the efficacy and tolerability of T at two different dosages was evaluated (10 mg or 100 mg IV every 2 weeks). An objective response was observed in 40% and 36% of patients who received the 10 mg and 100 mg dosage, respectively. At the time of publication, the median duration of response was not yet available; however, it was at least 6 months in 59% of patients and at least 9 months in 29% of patients. The PFS was 4.9 months and 3.9 months in the 10 mg and 100 mg group, respectively. The estimated OS at 9 months was 68% in the 10 mg arm and 66% in the 100 mg arm. The most common adverse events (AEs) were related to cytokine release syndrome (fever, hypotension, and hypoxia: 51% and 61% in the 10 mg and 100 mg group, respectively), loss of appetite, pyrexia, constipation, and anemia, but only 3% of patients discontinued treatment due to toxicity. The authors concluded that T at a dose of 10 mg every 2 weeks showed interesting activity with durable objective responses and promising survival outcomes in this heavily pretreated group of SCLC patients. The 10 mg dose of T was therefore chosen for further studies due to its efficacy and favorable toxicity profile [28].

Other targets being studied are those related to DNA repair and cell cycle pathways, such as CDK4/6 (cyclin-dependent kinase 4 and 6) inhibitors (trilaciclib), EZH2 (enhancer of zeste 2 polycomb repressive complex 2 subunit), and PARP (poly (ADP-ribose) polymerase) inhibitors (olaparib, velaparib) [13].

Additional single agents with demonstrated activity in second-line SCLC therapy include paclitaxel, docetaxel, vinorelbine, oral etoposide, and gemcitabine [29] (Table 1).

Another very promising area of research is the development of antibody–drug conjugates (ADCs) as a treatment approach, which consist of antibodies targeting specific tumor antigens, delivering cytotoxic drugs directly to cancer cells, and reducing side effects [30].

A subgroup analysis of 21 patients with refractory SCLC from a phase I/II trial evaluating the novel ADC Ifinatamab Deruxtecan showed an ORR of 52% and a median duration of response of 5.9 months with a good safety profile. Median OS was 9.9 months. A phase II study of patients with second- or third-line ED SCLC only is currently ongoing [31].

Usually, in ED SCLC, RT is used for symptomatic purposes. However, in those patients with good PS and who have achieved a complete response of extrathoracic disease, RT on the residual thoracic disease represents an option, as shown by the positive data from the phase III study by Slotman et al. [32]. Furthermore, similarly to LD SCLC, prophylactic cranial irradiation (PCI) should also always be considered for all patients who have achieved a good response [33,34]. Indeed, a randomized study of the European Organization for Research and Treatment of Cancer (EORTC) has highlighted how the 1-year OS doubles (27.1% versus 13.3%) in favor of those patients who had received PCI [35].

## 4. Discussion

Despite significant progress in second-line treatment options for SCLC, several challenges remain. First of all, patient selection represents a priority and it is mandatory to identify both the most appropriate second-line therapy for each individual patient and those biomarkers and predictive factors that can guide therapeutic decisions. Furthermore, SCLC is known for its ability to rapidly develop resistance to therapy. Therefore, understanding underlying resistance mechanisms and developing strategies to overcome them is a critical area of research. Third, many second-line treatments, particularly chemotherapy regimens, can be associated with significant toxicity that worsens patients’ QoL. Therefore, seeking treatments with a better side effect profile is a priority. Finally, although some second-line treatments have shown promise, overall data on their long-term efficacy and onfimpact on survival are still limited. Further researches are needed to refine treatment approaches.

The field of SCLC research is dynamic, with ongoing efforts to improve second-line therapeutic options. Some possible future directions include personalized medicine through developing personalized treatment plans based on the patient’s genetic and molecular profile; targeted therapies directed at specific genetic alterations, which may offer more effective treatment options; further exploration of combination therapies, including targeted agents and immunotherapies, which is likely to lead to more effective and less toxic treatments; efforts to detect SCLC at an early and more treatable stage, which has the potential to reduce the need for extensive second-line treatments; and identifying predictive biomarkers for treatment response, which will allow for more precise selection of patients for second-line treatments.

Finally, ongoing clinical trials continue to evaluating new agents and treatment combinations. These trials not only contribute to expanding the therapeutic armamentarium but also provide valuable data to refine treatment strategies and inform clinical practice.

## 5. Conclusions

Second-line treatment options for SCLC have evolved significantly in recent years, with a growing emphasis on personalized medicine, immunotherapy, and combination therapies. If it is not possible to include the patient in a clinical trial, which remains a preferential choice in this group of patients, the assessment of PS and PFS is mandatory for a therapeutic decision. In patients with PS > 2, supportive care only is recommended.

While challenges remain, ongoing research continues to contribute to improving therapeutic options and outcomes for patients with SCLC.
